# A novel conditional survival nomogram for monitoring real-time prognosis of non-metastatic colorectal cancer

**DOI:** 10.1007/s12672-024-01042-9

**Published:** 2024-05-21

**Authors:** Pei Luo, Ying-ying Li, Can Huang, Jun Guo, Xin Yao

**Affiliations:** 1 Department of Gastroenterology, People’s Hospital of Qianxinan Prefecture, Xingyi, Guizhou, 562400 China; 2 Department of Gerontology, People’s Hospital of Qianxinan Prefecture, Xingyi, Guizhou, 562400 China

**Keywords:** Colorectal cancer, SEER database, Conditional survival nomogram, Cancer prognosis

## Abstract

**Aims:**

The aim of this study is to enhance the accuracy of monitoring and treatment information for patients diagnosed with colorectal cancer (CRC).

**Methods:**

Utilizing the Surveillance, Epidemiology, and End Results (SEER) database, a cohort of 335,948 eligible CRC patients was included in this investigation. Conditional survival probability and actuarial overall survival were employed as methodologies to investigate the association between clinicopathological characteristics and cancer prognosis.

**Results:**

Among CRC patients, the 5-year survival rate was 59%, while the 10-year survival rate was 42%. Over time, conditional survival showed a consistent increase, with rates reaching 45% and 48% for individuals surviving 1 and 2 years, respectively. Notably, patients with unfavorable tumor stages exhibited substantial improvements in conditional survival, thereby narrowing the disparity with actuarial overall survival over time.

**Conclusion:**

This study underscores the significance of time-dependent conditional survival probability, particularly for patients with a poorer prognosis. The findings suggest that long-term CRC survivors may experience improved cancer prognosis over time.

## Introduction

Colorectal cancer (CRC), alternatively referred to as colon or rectal cancer, stands as a prevalent malignant ailment on a global scale. Positioned as the third most prevalent cancer and the second primary contributor to cancer-associated fatalities worldwide, CRC imposes a substantial burden [1, 2]. Despite notable strides in diagnostic and therapeutic realms, the task of prognosticating individual patient outcomes at distinct junctures remains inherently challenging [3]. Prognostic models, epitomized by survival nomograms, assume pivotal roles in foreseeing patient survival by leveraging specific clinical and pathological variables [4, 5]. However, conventional nomograms conventionally presuppose constant hazard rates over time, a premise that might inadequately capture the dynamic evolution of cancer progression and treatment response [6].

To overcome this inherent limitation, we propose a pioneering approach: a novel conditional survival nomogram tailored specifically for monitoring the real-time prognosis of non-metastatic colorectal cancer [7–9]. This CS-nomogram harnesses sophisticated statistical modeling techniques, its efficacy contingent upon the underlying assumptions established during its development [10, 11]. Crucially, rigorous validation of the nomogram's predictive accuracy is imperative, alongside an assessment of its applicability across diverse patient cohorts and healthcare settings [12, 13]. This innovative methodology aims to furnish personalized prognostic insights grounded in patient-specific attributes and the dynamic nature of the disease trajectory. By integrating time-varying covariates, we afford a systematic exploration of patient data at various temporal junctures, facilitating meticulous monitoring of prognosis and treatment efficacy [14]. Given the pronounced clinical heterogeneity and treatment responsiveness exhibited by colorectal cancer, precise prognostic appraisal assumes paramount significance for optimal patient care. Traditional survival nomograms, predicated upon baseline patient characteristics, regrettably, falter in encapsulating the temporal dynamics of prognostic determinants [15]. Our proposed conditional survival nomogram bridges this critical lacuna by encompassing dynamic variables such as treatment response, disease progression, and late-effects, thereby enabling real-time prognosis monitoring. Moreover, the utilization of nomograms confers several notable advantages over conventional survival analysis methodologies. Nomograms furnish a visually intuitive depiction of prognostic factors and their corresponding weights, bolstering the interpretability and clinical utility of predictive models [16]. Furthermore, they afford personalized risk assessments tailored to an individual patient's unique profile and disease trajectory. This personalized prognostic evaluation approach holds promise in guiding treatment decisions, enriching patient counseling endeavors, and ultimately augmenting long-term survival outcomes [17].

In summary, our study endeavors to pioneer the development of a groundbreaking conditional survival nomogram tailored specifically for monitoring real-time prognosis in non-metastatic colorectal cancer patients. This innovative methodology will meticulously incorporate time-varying covariates, thereby furnishing personalized prognostic insights to clinicians. By empowering clinicians with patient-specific characteristics and insights into the dynamic evolution of the disease, we aim to equip them with the tools necessary to make informed decisions. Through the implementation of this cutting-edge prognostic tool, we anticipate a transformative impact on patient outcomes, alongside a deeper understanding of the evolving nature of colorectal cancer. We are optimistic that this novel approach will contribute significantly to the advancement of personalized medicine in the realm of colorectal cancer care.

## Materials and methods

### Data source and study population

The data utilized for this study were sourced from the Surveillance, Epidemiology, and End Results (SEER) database, accessible via https://seer.cancer.gov/. Renowned for its comprehensive repository of cancer patients spanning diverse geographic regions within the United States, the SEER database offers a wealth of meticulously documented clinical and epidemiological information [18]. Our study focused on patients diagnosed with colorectal cancer within a specified timeframe. Inclusion criteria encompassed histologically confirmed primary colorectal cancer cases, coupled with the availability of comprehensive demographic, clinical, and follow-up data. Furthermore, TNM staging was redefined in accordance with the eighth edition of TNM staging guidelines, accounting for tumor size, lymph node metastasis, and distant tumor metastasis. Patients with incomplete or missing data were excluded from the analysis, ensuring the integrity and robustness of our dataset.

### Ethical considerations

This study rigorously adhered to the guidelines and regulations stipulated by the SEER database. Given that the SEER database houses de-identified patient information, ethical approval or informed consent was not deemed necessary for this research endeavor.

### Variables of interest

The selection of clinical and pathological variables for analysis was guided by their established association with colorectal cancer prognosis. These variables comprised age at diagnosis, sex, race/ethnicity, tumor location, tumor stage, histological subtype, tumor grade, and treatment modalities (including surgery, chemotherapy, and radiation therapy). Additionally, pertinent data pertaining to vital status and survival duration were meticulously collected to facilitate the computation of conditional survival probabilities.

### Development of the conditional survival nomogram

To construct the conditional survival nomogram, we employed a robust statistical modeling approach. Initially, least absolute shrinkage and selection operator (LASSO) regression analysis was conducted to identify significant prognostic factors associated with survival outcomes. Time-dependent covariates were integrated to capture the dynamic nature of prognostic factors over time. Subsequently, the identified prognostic factors were utilized to formulate the conditional survival nomogram. Conditional survival rate denotes the probability that a colorectal cancer patient will survive y years subsequent to surviving x years from the date of diagnosis, expressed mathematically as CS(y|x) = OS(x + y)/OS(x), where CS(y|x) represents the probability of the patient being alive for x + y years post-diagnosis, and x denotes successful survival for x years. Each variable’s contribution to the overarching prognostic model was quantified using hazard ratios (HR) alongside corresponding 95% confidence intervals (CI). Covariate coefficients were employed to allocate points on the nomogram's scale. The cumulative sum of these points for each patient facilitated the determination of their individualized probability of survival at specific time intervals.

### CS-nomogram model accuracy

To evaluate the performance and accuracy of the conditional survival nomogram, we adopted rigorous internal validation techniques including bootstrapping and cross-validation. These methods were instrumental in assessing the nomogram's calibration and discrimination capabilities, ensuring its reliability in predicting survival probabilities over time. Furthermore, we conducted comparative analyses to juxtapose the performance of the conditional survival nomogram against that of traditional survival models, such as the TNM staging system. This enabled us to gauge the superiority of our proposed nomogram in prognostic accuracy and clinical utility, providing valuable insights into its potential as a robust prognostic tool for non-metastatic colorectal cancer patients.

### Statistical analysis

The statistical analysis was conducted using R software version 4.3.5. Patient demographics and clinical characteristics were summarized using descriptive statistics. Survival analysis techniques, such as Kaplan–Meier estimation and log-rank tests, were employed to assess survival outcomes across different subgroups. LASSO regression models were utilized to identify prognostic factors associated with colorectal cancer outcomes. The development and validation of the conditional survival nomogram will be detailed, emphasizing its predictive accuracy and clinical applicability. The Materials and Methods section comprehensively outlined the study design, data source, patient selection criteria, variables of interest, development of the conditional survival nomogram, model validation procedures, ethical considerations, and statistical analysis methods. These methodologies were meticulously implemented to capture the dynamic nature of colorectal cancer prognosis using patient data from the SEER database.

## Results

### Patient demographics

**Fig. 1:**
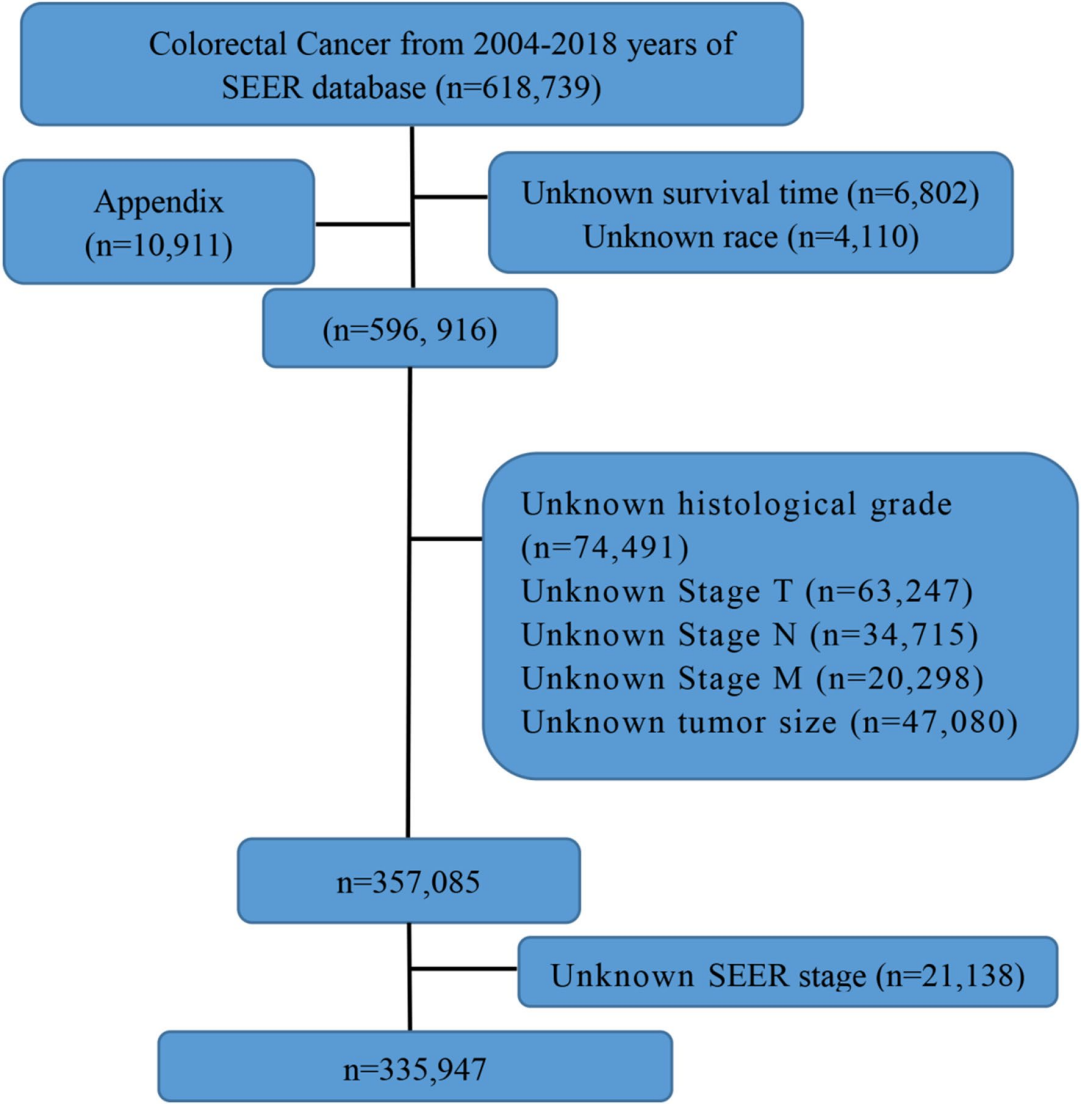
Guidelines for colorectal cancer patient screening: A SEER (Surveillance, Epidemiology, and End Results) program flowchart

Our study analyzed data from 335,948 colorectal cancer (CRC) patients registered in the SEER database from years ranging 2004 to 2018. The median age at diagnosis was 66.6 years old. The ratio composition of thepatient population was 79.9% white, 11.3%black, and 8.8% from other racial backgrounds. Regarding the age population, 3.3% of patients under 40 years old, 53.3% fell within the 40–70 years old range, and 43.4% over 70 years old (Table 1, Fig. 1).

**Table 1:** Clinicopathologic characteristics of colorectal patients

Characteristics	Level	Whole cohort	Training group	Validation group	p
n		n = 325,773 (%)	n = 228,041 (%)	97,732	
Age at diagnosis, years (mean(SD))		66.6 (47.5)	66.5 (47.5)	66.6 (47.6)	0.819
Age	≤ 40	10,713 (3.3)	7502 (3.3)	3211 (3.3)	0.892
	41–70	173,736 (53.3)	121,553 (53.3)	52,183 (53.4)	
	≥ 71	141,324 (43.4)	98,986 (43.4)	42,338 (43.3)	
Marital status	Unmarried	148,540 (45.6)	104,055 (45.6)	44,485 (45.5)	0.557
	Married	177,233 (54.4)	123,986 (54.4)	53,247 (54.5)	
Race	White	260,454 (79.9)	182,254 (79.9)	78,200 (80.0)	0.814
	Black	36,726 (11.3)	25,756 (11.3)	10,970 (11.2)	
	Other	28,593 (8.8)	20,031 (8.8)	8562 (8.8)	
T status (AJCC 8th)	I	58,309 (17.9)	40,761 (17.9)	17,548 (18.0)	0.862
	II	48,869 (15.0)	34,197 (15.0)	14,672 (15.0)	
	III	170,931 (52.5)	119,759 (52.5)	51,172 (52.4)	
	IV	47,664 (14.6)	33,324 (14.6)	14,340 (14.7)	
N status (AJCC 8th)	N0	194,555 (59.7)	136,309 (59.8)	58,246 (59.6)	0.048
	N1	81,781 (25.1)	57,355 (25.2)	24,426 (25.0)	
	N2 or N3	49,437 (15.2)	34,377 (15.1)	15,060 (15.4)	
M status (AJCC 8th)	M0	279,275 (85.7)	195,414 (85.7)	83,861 (85.8)	0.394
	M1	46,498 (14.3)	32,627 (14.3)	13,871 (14.2)	
Histological grade	I	33,290 (10.2)	23,411 (10.3)	9879 (10.1)	0.133
	II	228,761 (70.2)	160,199 (70.3)	68,562 (70.2)	
	III or IV	63,722 (19.6)	44,431 (19.5)	19,291 (19.7)	
Type of surgery	No surgery	16,732 (5.1)	11,718 (5.1)	5014 (5.1)	0.897
	Local ablation or excision	16,259 (5.0)	11,355 (5.0)	4904 (5.0)	
	Resection of partial or whole organ	292,782 (89.9)	204,968 (89.9)	87,814 (89.9)	
Number of regional lymph node resection	0	40,596 (12.5)	28,373 (12.4)	12,223 (12.5)	0.703
	1–7	10,712 (3.3)	7531 (3.3)	3181 (3.3)	
	8 +	274,465 (84.3)	192,137 (84.3)	82,328 (84.2)	
Radiotherapy	No	281,902 (86.5)	197,303 (86.5)	84,599 (86.6)	0.755
	Yes	43,871 (13.5)	30,738 (13.5)	13,133 (13.4)	
Chemotherapy	No	199,846 (61.3)	139,914 (61.4)	59,932 (61.3)	0.867
	Yes	125,927 (38.7)	88,127 (38.6)	37,800 (38.7)	
Regional lymph node positive	Negative	168,688 (51.8)	118,146 (51.8)	50,542 (51.7)	0.625
	Positive	157,085 (48.2)	109,895 (48.2)	47,190 (48.3)	
Number of tumors	1	227,124 (69.7)	159,040 (69.7)	68,084 (69.7)	0.583
	2	73,511 (22.6)	51,359 (22.5)	22,152 (22.7)	
	3+	25,138 (7.7)	17,642 (7.7)	7496 (7.7)	

*AJCC-8th* American Joint Committee on Cancer (8th Edition); TNM, *SD* standard deviation

### Overall survival and conditional survival analysis

**Fig. 2:**
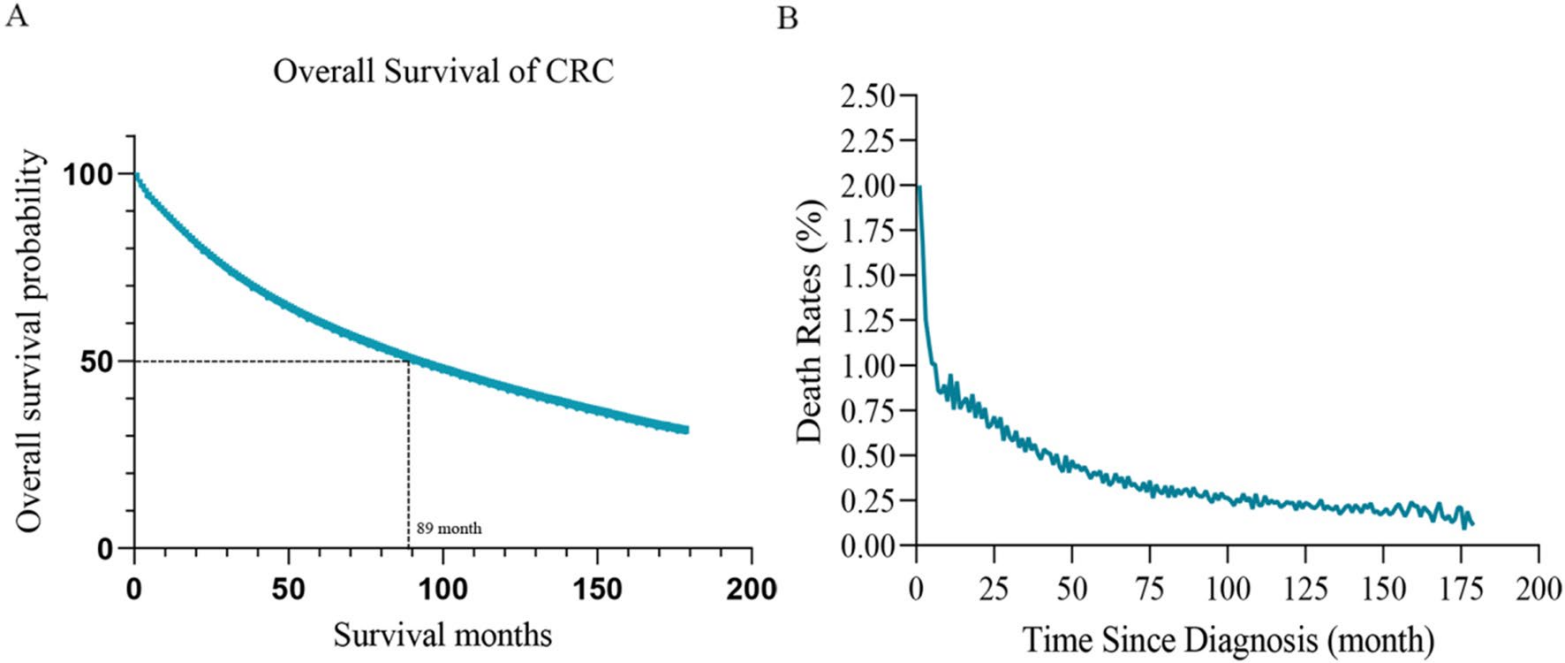
Survival analysis: overall survival (**A**) and monthly mortality rates (**B**) for the entire patient cohort

**Fig. 3:**
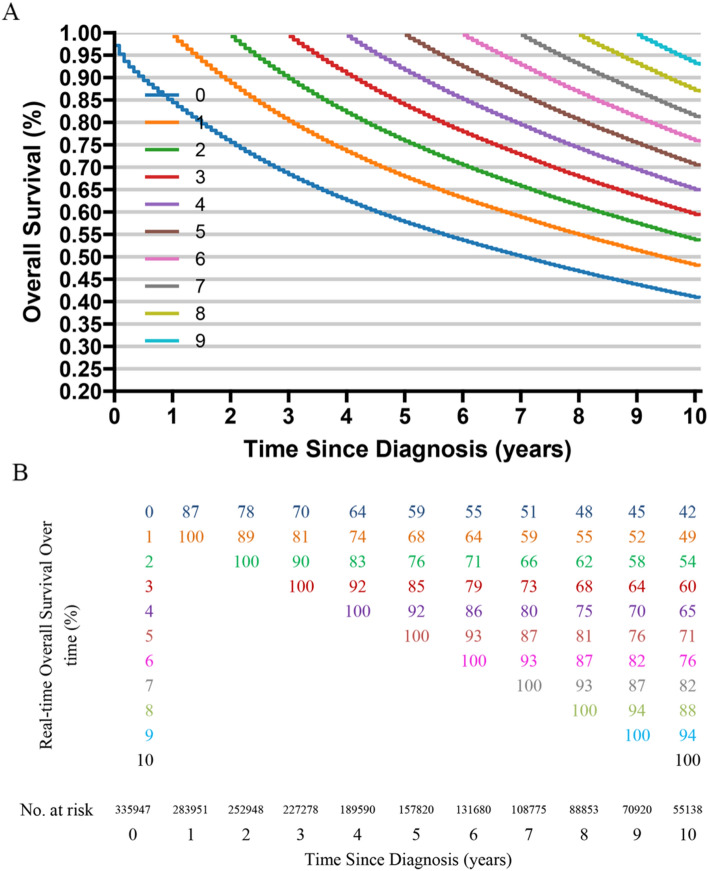
Kaplan–Meier method for estimating conditional cancer survival (C-CS) at 10 years after surviving 0–10 years in colorectal cancer patients. Conditional survival curves (**A**) and their updated survival data adjusted for survived time (**B**)

The median survival time for CRC patients was 89 months (Fig. 2A). Utilizing Kaplan–Meier analysis, we observed that the 5-year survival rate for CRC patients stood at 59% (95% CI 59–59%), while the 10-year survival rate was 42% (95% CI 42–42%). Additionally, our conditional survival probability analysis unveiled a progressive increase in the 10-year overall survival rate with each additional year since diagnosis. For instance, among patients surviving 1 and 2 years post-diagnosis, the corresponding 10-year survival rates (representing the probability of living another 9 and 8 years) were determined to be 45% and 48%, respectively. Similarly, patients who survived 3–9 years from the date of diagnosis exhibited escalating 10-year survival rates of 51%, 55%, 59%, 64%, 70%, 78%, and 87%, respectively (Table 2, Fig. 3).

**Table 2:** Probability of patients with CRC patients can reach a certain survival time after a period of survival

Years already survived									
Survival probability to reach X years Time	0 Year	1 Year	2 Year	3 Year	4 Year	5 Year	6 Year	7 Year	8 Year
1 Year	0.87 (0.87–0.87)	1							
2 Year	0.78 (0.78–0.78)	0.89 (0.89–0.90)	1						
3 Year	0.70 (0.70–0.70)	0.81 (0.81–0.81)	0.90 (0.90–0.91)	1					
4 Year	0.64 (0.64–0.64)	0.74 (0.74–0.74)	0.83 (0.83–0.83)	0.92 (0.91–0.92)	1				
5 Year	0.59 (0.59–0.59)	0.68 (0.68–0.68)	0.76 (0.76–0.77)	0.85 (0.84–0.85)	0.92 (0.92–0.92)	1			
6 Year	0.55 (0.55–0.55)	0.64 (0.63–0.64)	0.71 (0.71–0.71)	0.79 (0.78–0.79)	0.86 (0.86–0.86)	0.93 (0.93–0.93)	1		
7 Year	0.51 (0.51–0.51)	0.59 (0.59–0.59)	0.66 (0.66–0.66)	0.73 (0.73–0.73)	0.80 (0.80–0.80)	0.87 (0.86–0.87)	0.93 (0.93–0.93)	1	
8 Year	0.48 (0.48–0.48)	0.55 (0.55–0.56)	0.62 (0.62–0.62)	0.68 (0.68–0.69)	0.75 (0.74–0.75)	0.81 (0.81–0.81)	0.87 (0.87–0.87)	0.93 (0.93–0.94)	1
9 Year	0.45 (0.45–0.45)	0.52 (0.52–0.52)	0.58 (0.58–0.58)	0.64 (0.64–0.64)	0.70 (0.70–0.70)	0.76 (0.76–0.76)	0.82 (0.81–0.82)	0.87 (0.87–0.88)	0.94 (0.93–0.94)
10 Year	0.42 (0.42–0.42)	0.48 (0.48–0.49)	0.54 (0.54–0.54)	0.60 (0.60–0.60)	0.65 (0.65–0.66)	0.71 (0.71–0.71)	0.76 (0.76–0.77)	0.82 (0.82–0.82)	0.88 (0.87–0.88)
Number at risk	*335,947*	*283,951*	*252,948*	*227,278*	*189,590*	*157,820*	*131,680*	*108,775*	*88,853*

Furthermore, our analysis of the mortality rate and annual hazard rate revealed that initial diagnosis was associated with a mortality rate of 1.999%. However, upon surviving the first year post-diagnosis, patients experienced a notable decline in their average mortality rate to 0.754%. This downward trend persisted, with further reductions observed in the third year, fifth year, and eighth year, resulting in average mortality rates of 0.598%, 0.347%, and 0.289%, respectively (Fig. 2B). Moreover, the annual risk rate exhibited a non-linear relationship, with the highest risk occurring in the first year following diagnosis. However, as patients surpassed the initial year milestone, their probability of death gradually diminished, underscoring the dynamic nature of mortality risk over time.

### Overall survival and conditional survival analysis based on TNM stage

We identified a significant correlation between TNM stage and prognosis among CRC patients. Specifically, the 3-year survival rates for Stage I, II, III, and IV were determined to be 85%, 78%, 70%, and 28%, respectively. Our conditional survival analysis revealed that the 3-year survival rates for patients with different TNM stages approached each other as the survival time increased. For patients who survived up to the fifth year after diagnosis, the probabilities of surviving to the eighth year were 84%, 80%, 80%, and 63% for Stage I, II, III, and IV, respectively. Moreover, we observed distinct fluctuations in survival rates based on specific factors. For instance, patients diagnosed with pathological T1 stage CRC exhibited an initial 3-year survival rate of 80%, which escalated to 86% if they survived up to the fifth year post-diagnosis. Analogous trends were discerned among patients stratified by other stage-specific criteria (Table 3).

**Table 3:** 1-, 3-, 5-, 8-year OS rate sand 3-year conditional survival rates of patients in relationship to clinicopathologic features

Variables	1-, 3-, 5-, 10-year OS rates, (%)				3-year conditional survival probability (%)				
	1-year OS	3-year OS	5-year OS	10-year OS	1 y after diagnosis	2 y after diagnosis	3 y after diagnosis	4 y after diagnosis	5 y after diagnosis
All patients	87	70	59	42	74	76	79	80	81
Age									
≤ 40 years	93	78	70	62	79	83	86	89	91
41–70 years	91	77	68	55	79	82	84	86	88
≥ 71 years	81	61	48	25	67	69	70	70	69
Race (%)									
White	87	70	59	42	74	76	78	80	80
Black	85	66	55	40	70	74	77	80	81
Other	90	75	65	51	77	79	82	84	85
Marital status									
Married	89	74	64	48	77	79	81	82	83
Unmarried or divorce or death of spouse	84	65	54	35	70	73	75	77	78
8th AJCC pathological stage (%)									
Stage I	93	85	76	56	86	86	85	85	84
Stage II	91	78	67	46	79	79	79	80	80
Stage III	88	70	58	41	72	74	77	79	80
Stage IV	64	28	15	8	31	36	45	54	63
Pathological T stage (%)									
pT1	90	80	73	56	84	86	86	86	86
pT2	93	83	73	53	84	83	83	82	82
pT3	88	70	58	40	72	74	76	78	79
pT4	73	45	33	22	53	60	67	72	76
Pathological N stage (%)									
pN0	91	78	68	49	81	81	81	82	82
pN1	85	66	54	38	69	72	76	78	80
pN2-3	75	46	34	23	52	59	65	71	76
Pathological M stage (%)									
pM0	91	77	67	48	79	80	80	81	82
pM1	64	28	15	8	31	36	45	54	63
Histological grade (%)									
Grade I	92	81	71	53	82	83	83	84	84
Grade II	89	73	62	44	75	77	78	80	81
Grade III and IV	76	55	45	31	65	72	76	78	80
Type of surgery									
Resection of partial or whole organ	88	72	61	43	75	77	79	80	81
Local ablation or excision	93	82	72	54	83	83	84	84	84
No surgery	59	30	21	14	41	52	63	71	75
Radiation									
Yes	90	73	61	44	73	75	78	80	81
No	86	70	59	42	74	77	79	80	81
Chemotherapy									
Yes	89	68	56	42	69	73	77	80	82
No	85	71	61	42	77	79	80	80	80
Regional lymph node metastasis									
Without lymph node metastasis	93	81	71	51	82	82	82	82	82
Lymph node metastasis	81	58	47	33	64	69	74	77	79
Number of tumors									
Single tumor	87	71	61	46	75	78	81	82	84
Two tumors	86	69	57	36	72	74	75	76	76
Three and above tumors	87	68	54	30	70	71	71	71	71

### Overall survival and conditional survival analysis based on type of surgery

Based on the type of surgery, we categorized patients into three subgroups: (1) non-surgical, (2) local ablation or excision, and (3) resection of partial or whole organ. The 3-year survival rates for these groups were 30%, 82%, and 72%, respectively, while the 10-year survival rates were 14%, 54%, and 43%. For patients who survived 5 years after diagnosis, the probabilities of still being alive at 8th year were 75%, 84%, and 81% for the three subgroups. The 3-year survival rate for patients who received radiation therapy was 73%, indicating an 81% chance of survival at 8th year if they lived up to the fifth year after diagnosis. We also observed varying changes in survival rates based on the provision of radiation and chemotherapy (Table 3).

### LASSO regression analyses

**Fig. 4:**
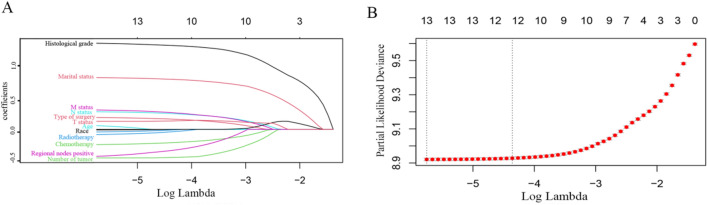
Predictor screening with LASSO regression and 10-fold cross-validation (**A**) and (**B**)

We performed LASSO regression analysis to discern significant prognostic factors associated with survival outcomes in CRC patients. This analysis unveiled several variables that exerted a substantial impact on overall survival. These variables encompassed age, marital status, race, pathological stage (T, N, or M), histological grade, type of surgery, resection of lymph nodes, radiation therapy, chemotherapy, regional nodes positivity, and the number of tumors (Fig. 4).

### Development of the conditional survival nomogram

**Fig. 5:**
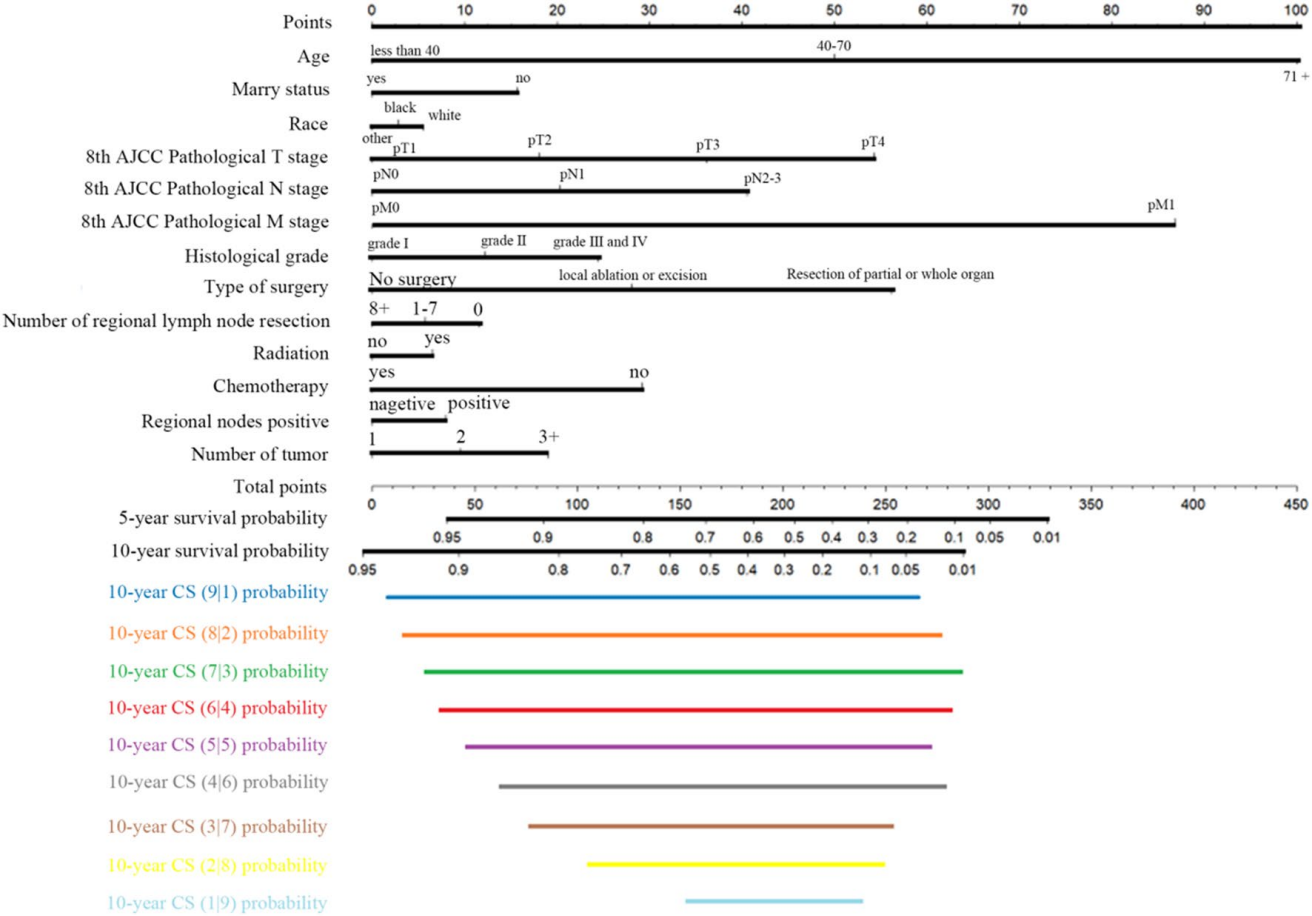
CS-nomogram for predicting 5-year, 10-year, and 10-year conditional cancer-specific survival (C-CSS) in colorectal cancer patients

Using the identified significant prognostic factors, we developed a novel conditional survival nomogram for CRC patients. This nomogram incorporated time-dependent covariates to capture dynamic changes in prognostic variables over time. By providing individualized survival probabilities at specific time intervals, the nomogram enables real-time prognosis monitoring. The nomogram is based on a scoring system that considered the impact of each included factor on overall survival prognosis. By combining the conditional survival rate formula with the nomogram, we created a conditional survival nomogram (CS-nomogram) that predicts the probability of each patient surviving y years after living x years, based on their clinicopathological parameters. For instance, a patient with a nomogram total score of 180 has a 10-year survival rate of 42%. If this patient is still alive at the third year after diagnosis, the probability of still being alive at the 10th year after diagnosis is 69% (Fig. 5).

## Discussion

The development of a novel conditional survival nomogram for monitoring real-time prognosis in CRC patients marks a significant milestone in the field, as corroborated by previous studies [12, 19, 20]. This personalized tool integrates time-varying covariates and patient-specific characteristics to furnish individualized survival estimates at specific time intervals, thereby bolstering the foundation for tailored treatment decision-making [21, 22]. By offering real-time prognostic information, the nomogram serves as a valuable resource in patient counseling and shared decision-making processes, facilitating the dissemination of tailored survival estimates at distinct time points [23]. This enhanced communication fosters an environment conducive to alleviating patient anxiety, fostering realistic expectations, and empowering patients to actively engage in their care journey [24].

The analysis results underscored a 5-year survival rate of 59%, indicative of a moderate prognosis for CRC patients. However, the 10-year survival rate exhibited a decline to 42%, signaling a lower long-term survival outlook for these individuals. This underscores the imperative of timely diagnosis, efficacious treatment, and comprehensive long-term management strategies for CRC patients. Additionally, the conditional survival analysis provided valuable insights into the dynamic changes in survival rates over time. The probabilities of surviving for an additional 9 or 8 years increased to 45% and 48%, respectively, if patients lived another 1 or 2 years after their initial diagnosis. Notably, the overall 10-year survival rates significantly improved for patients who survived beyond the first year, with rates ranging from 51 to 87% depending on the number of years survived. This indicates that survival rates progressively improve as patients achieve longer-term remission.

The correlation between TNM stage and survival outcomes was strikingly evident in our analysis. Stage IV CRC patients exhibited the lowest 3-year survival rate of merely 28%, while those classified as stage I patients demonstrated the highest rate at 85%. However, as survival time extended, the survival rates for all stages progressively converged. This phenomenon suggests that regardless of the initial TNM stage, survival prospects may tend to become more similar over time. The type of surgery emerged as a pivotal determinant of survival rates. Non-surgical patients manifested the lowest 3-year survival rate of 30%, whereas individuals undergoing local ablation or excision and resection of partial or whole organs showcased substantially superior rates of 82% and 72%, respectively. Further analysis unveiled that patients who received radiation therapy exhibited a relatively higher 3-year survival rate of 73%, hinting at the potential benefits associated with this treatment modality. These observations underscore the pivotal role of surgical intervention and hint at the potential efficacy of radiation therapy in enhancing survival outcomes for CRC patients. Furthermore, the LASSO regression analysis unveiled several factors significantly influencing overall survival, including age, marital status, race, pathological stage, histological grade, type of surgery, lymph node resection, radiation therapy, chemotherapy, and tumor-related variables. These findings emphasize the multifactorial nature of CRC prognosis and underscore the imperative of considering multiple variables in clinical decision-making and prognosis estimation.

The CS-nomogram offers several noteworthy clinical implications. Firstly, it enables oncologists and clinicians to monitor patients' prognosis throughout the disease trajectory [13, 25, 26]. By considering dynamic variables such as treatment response, disease progression, and late-effects, the incorporation of dynamic variables in the nomogram allows for timely identification of patients at high risk of disease recurrence or progression [27]. This early risk stratification enables clinicians to intensify follow-up measures, implement adjuvant therapies, or consider alternative treatment approaches [28, 29]. Early intervention based on accurate prognostic information can potentially improve survival outcomes and enhance disease management. In the same time, there are certain limitations that need to be acknowledged. Firstly, this study utilized retrospective data from the SEER database, which may introduce inherent biases. The accuracy and reliability of the CS-nomogram's predictive performance depend on the quality of the data source and the completeness of the collected variables [30, 31]. Prospective studies with larger sample sizes and robust data collection methods are warranted to further validate the nomogram's clinical utility. Another limitation is the lack of inclusion of certain important prognostic factors, such as comorbidities and molecular biomarkers. The assessment of these variables could potentially enhance the accuracy and predictive power of the nomogram. Future research should explore incorporating these additional factors into the model to further improve prognostic accuracy [32].

FContinual refinement and enhancement of the conditional survival nomogram for CRC are imperative to keep pace with evolving clinical needs. One avenue for improvement involves incorporating emerging biomarkers, such as genetic mutations and microenvironment characteristics, into the model. By integrating these factors, a more comprehensive understanding of tumor biology can be attained, facilitating the development of individualized treatment strategies tailored to patients' specific molecular profiles. Moreover, prospective studies featuring long-term follow-up are indispensable for evaluating the sustained predictive value and clinical impact of the conditional survival nomogram. These investigations can furnish real-world evidence regarding the nomogram's efficacy in guiding treatment decisions, enhancing patient outcomes, and optimizing resource allocation in healthcare settings. The potential integration of machine learning algorithms and artificial intelligence techniques holds promise for further enhancing the accuracy and precision of the nomogram. Leveraging big data analytics and advanced computational methods enables the development of more sophisticated prediction models capable of capturing subtle changes in the disease course and treatment response, thereby facilitating personalized and proactive patient care. Continued research efforts in these directions are pivotal for advancing the field of CRC prognosis prediction and improving patient management strategies.

## Conclusion

The development of a novel conditional survival nomogram for non-metastatic colorectal cancer represents a promising advancement in prognosis monitoring. This personalized tool, incorporating time-varying covariates and patient-specific characteristics, offers real-time prognostic information for individual patients. While certain limitations need to be addressed, the nomogram holds great potential to guide treatment decisions, improve patient counseling, and enhance long-term survival outcomes. Further research and validation are warranted to fully realize the clinical utility of this innovative approach.

## Data Availability

All data generated or analyzed during this study are included in this published article or are available from the corresponding author on reasonable request. The dataset generated and analyzed during the current study is available in the Surveillance Epidemiology and End Results (SEER) Database repository (https://seer.cancer.gov/).
